# The elevation of fibroblast growth factor 21 is associated with generalized periodontitis in patients with treated metabolic syndrome

**DOI:** 10.1186/s12903-022-02533-3

**Published:** 2022-12-06

**Authors:** Teerat Sawangpanyangkura, Panwadee Bandhaya, Pattanin Montreekachon, Anongwee Leewananthawet, Arintaya Phrommintikul, Nipon Chattipakorn, Siriporn C. Chattipakorn

**Affiliations:** 1 Department of Restorative Dentistry and Periodontology, Faculty of Dentistry, Chiang Mai University, Chiang Mai, 50200 Thailand; 2 Department of Internal Medicine, Faculty of Medicine, Chiang Mai University, Chiang Mai, Thailand; 3 Neuroelectrophysiology Unit, Cardiac Electrophysiology Research and Training Center, Faculty of Medicine, Chiang Mai University, Chiang Mai, 50200 Thailand; 4 Center of Excellence in Cardiac Electrophysiology Research, Chiang Mai University, Chiang Mai, 50200 Thailand; 5 Department of Oral Biology and Diagnostic Sciences, Faculty of Dentistry, Chiang Mai University, Chiang Mai, 50200 Thailand

**Keywords:** FGF21, periodontitis, Metabolic syndrome

## Abstract

**Background:**

Fibroblast growth factor 21 (FGF21) is closely associated with metabolic syndrome (MetS). An alteration of FGF21 is possibly affected by periodontitis. The present study aimed to investigate the levels of serum FGF21 in MetS patients with generalized periodontitis and its association with periodontal and metabolic parameters.

**Methods:**

One hundred forty-six MetS patients were recruited from the CORE (Cohort Of patients at a high Risk for Cardiovascular Events) Thailand registry. All participants received general data interviewing, periodontal examination and blood collection for measurement of FGF21 levels and biochemistry parameters. Periodontitis was defined according to the new classification and divided into two groups of localized periodontitis and generalized periodontitis.

**Results:**

FGF21 was significantly higher in generalized periodontitis group when compared with localized periodontitis group (*p* <  0.05). The significant correlation was observed between FGF21 and variables including number of remaining teeth, mean clinical attachment loss, hypertriglyceridemia and low high-density lipoprotein cholesterol. The elevation of serum FGF21 was associated with presence of generalized periodontitis after adjusting of covariate factors (OR = 27.12, *p* = 0.012).

**Conclusions:**

The elevation of serum FGF21 might be a potential biomarker for MetS patients who have risk of generalized periodontitis.

## Background

Fibroblast growth factor 21 (FGF21) is a small molecular weight polypeptide of FGF hormone which is mainly produced in liver [1]. and also expressed in extrahepatic tissues such as adipose tissue and skeletal muscle [2]. FGF21 plays a crucial role in maintaining tissue homeostasis of both glucose and lipid metabolism [3] and responding to various stressful stimuli, such as inflammation [4], oxidative stress and hypoxia [5]. Previous studies showed that the elevation of circulating FGF21 was closely associated with metabolic disorders including obesity, diabetes and metabolic syndrome (MetS) [3, 6].

MetS is defined as a group of metabolic abnormalities including central obesity, hypertriglyceridemia, low levels of high-density lipoprotein cholesterol (HDL-C), hypertension, and an increased fasting plasma glucose level. The studies proposed the significance of MetS as a risk factor of diabetes and cardiovascular disease (CVD) [7, 8]. An increase of circulating FGF21 in MetS patients was hypothesized as a protective role against to metabolic stress or a compensatory upregulation to FGF21-resistance in tissues induced by obesity [3]. Up to date, multiple findings suggest that FGF21 could be a potential biomarker or promising therapeutic target for MetS [9–12]. In addition, one of the major oral problems which is commonly found in MetS patients is periodontitis [13, 14]. Periodontitis is an inflammatory disease caused by chronic accumulation of dental plaque, leading to the destruction of tooth-supporting tissue and tooth loss [15]. The evidences show that a state of systemic inflammation induced by periodontitis is a major key linking periodontitis to several diseases such as diabetes, CVD and Mets [14, 16, 17]. The several biomarkers linking periodontitis and MetS have been proposed. FGF21 is one of protein molecules that potentially involves the inflammatory and immunity response which is a fundamental process in pathogenesis of MetS and periodontitis. Therefore, FGF21 might be a potential biomarker for linking Mets to periodontitis.

Recently, there have been few attempts determining the level of FGF21 in periodontitis patients. One study showed the higher level of FGF21 in gingiva crevicular fluid (GCF) was collected from diabetic patients combined with periodontitis than those without periodontitis [18]. Moreover, after receiving periodontal treatment, the level of FGF21 in GCF was decreased, suggestive the anti-inflammation effect of FGF21 [18]. However, the other studies measuring FGF21 level in serum found that diabetic group demonstrated increased FGF21 following periodontal therapy as a result of improved glycemic control [19, 20]. These controversial outcomes were possibly because of different sources of FGF21 sampling, insufficient sample size to reach the power of a statistical test, various definition of periodontitis cases and no periodontally healthy control group or comparison groups such as localized and generalized periodontitis. Although the studies report contradictory findings, the expression of serum FGF21 in MetS patients might be related to periodontitis-induced inflammation. Up to date, there are no studies investigating the level of circulating FGF21 in patients with MetS and periodontitis. Therefore, the present study aimed to investigate the level of serum FGF21 in MetS patients with or without generalized periodontitis and the association of FGF21 to metabolic and periodontal parameters.

## Materials and methods

**Fig. 1:**
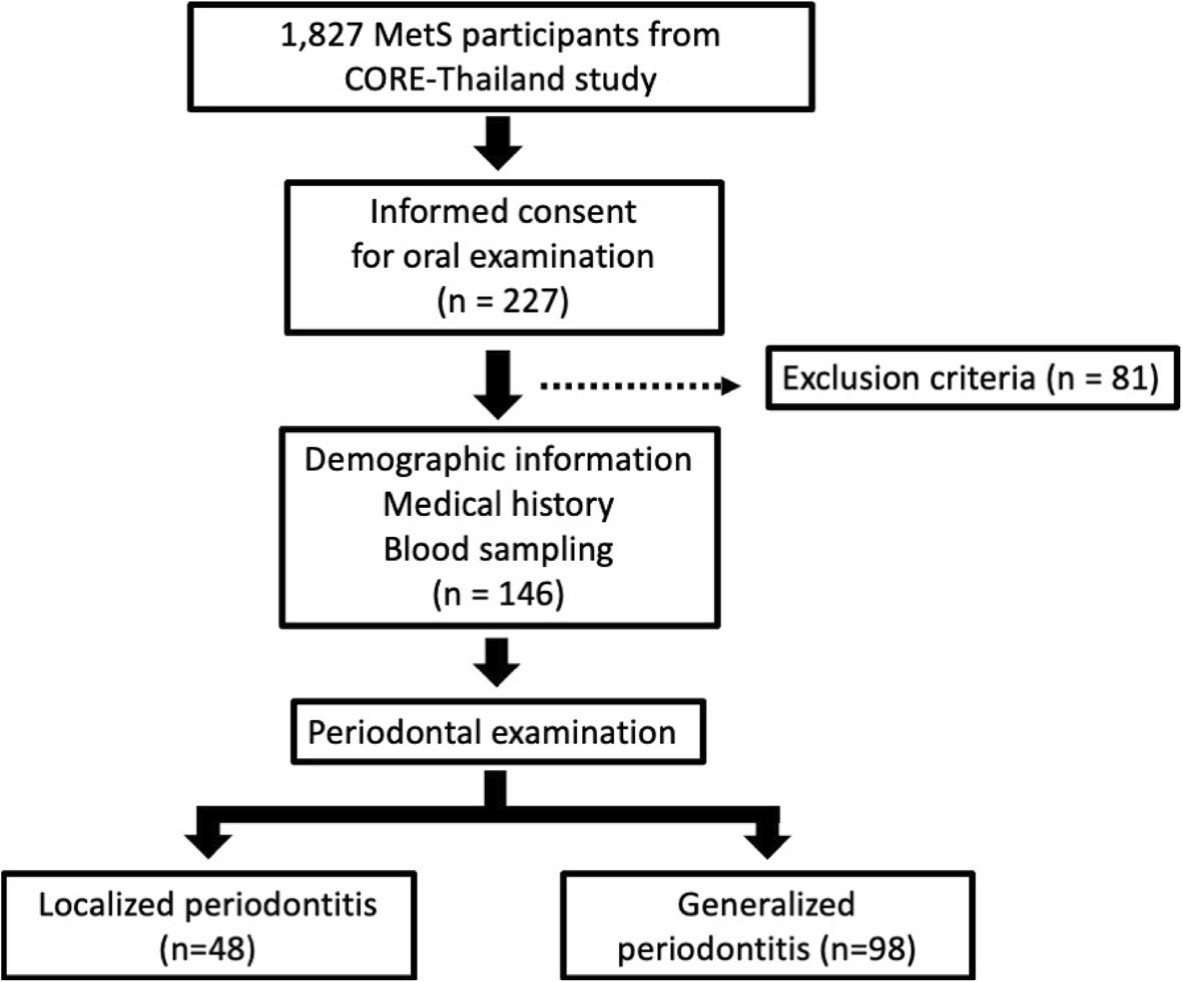
Schematic illustrating the flow of participant enrollment in the present study

This cross-sectional study was reviewed and approved by the institutional Human Experimentation Committee of the Faculty of Dentistry, Chiang Mai University, Chiang Mai, Thailand (Ethical approval number: 49/2016). Written informed consents were obtained from all participants on the date of recruitment. All methods were performed in accordance with the relevant guidelines and regulations. The present study was a sub-study of MetS patients in The Cohort of patients at a high Risk for Cardiovascular Events (CORE) - Thailand registry. The CORE-Thailand registry was an ongoing prospective study involving a cohort of Thai patients with high atherosclerotic risks. Patients with MetS undergoing medical treatment were enrolled from the outpatient clinic at Maharaj Nakorn Chiang Mai Hospital in the period October 2015 to April 2016. The criteria used for MetS diagnosis was according to a joint interim statement took place in 2009 [21]. The flow of participant recruitment was shown in Fig. 1.

Patients willing to receive periodontal examination were included and having the following conditions were excluded from the study: (1) antibiotic prophylaxis needed before periodontal examination; (2) received joint replacement surgery in last 2 years or present history of joint replacement infection; (3) present chronic kidney disease with undergoing hemodialysis; (4) having immunocompromised condition or immunodeficiency; (5) having bleeding disorders or receiving anticoagulants exception for aspirin with concentration under 325 mg/day.

### Data collection and sampling

Patient medical history collection, physical examination, fasting blood sampling and periodontal examination were performed on the date of enrollment. Blood samples of all participants were assessed for the calculation of the fasting blood sugar (FBS), HDL-C, LDL-C (low-density lipoprotein cholesterol), triglyceride, insulin and FGF21 levels. Serum FGF21 levels were determined using a human FGF21 enzyme-linked immunosorbent assay (ELISA) kit (R&D systems Inc., Minneapolis, MN, USA).

### Periodontal examination

The data including number of remaining teeth without third molars, plaque index (PI), gingival index (GI), probing pocket depth (PPD) and clinical attachment loss (CAL) were recorded. PI and GI indices were determined as described in a previous study [22]. For PPD and CAL, the full mouth examination was evaluated in six sites per tooth with a periodontal probe (PCPUNC 15, Hu-friedy, Chicago, IL, USA). Three calibration experts in periodontics performed a full mouth periodontal examination. Inter-examiner calibration was performed through the intraclass correlation coefficient with higher than 0.8 of the values for PPD and CAL were proven.

### Definition of periodontitis

Periodontitis is diagnosed as presence of detectable interdental CAL at two non-adjacent teeth and full-mouth BOP percentage ≥ 10%, according to new classification of periodontal disease. Moreover, the severity of disease was categorized into 4 stages with two levels of distribution including localized (< 30%) and generalized (≥30%) of affected teeth [23].

### Statistical analysis

Normality of data was analyzed by Kolmogorov-Smirnov test. Bivariate analysis was performed by unpaired T-test and Mann-Whitney U test for continuous variables. The comparison of nominal data between groups was performed by Chi-square test. The post hoc power based on FGF21 level was 0.96 with the effect size of 0.66. The Spearman rank was used to test correlation between continuous parameters. The association between the presence of generalized periodontitis and the serum FGF21 level was examined using multiple logistic regression analysis. The confounding factors that might affect periodontitis and FGF21 including age, gender, smoking, alcohol consumption, diabetic status, presence of component FBS, TG, HDL were adjusted. The analysis was conducted by SPSS version 17 (IBM Corp., Armonk, NY, USA).

## Results

146 MetS participants were recruited in the present study. Most subjects (87%) were classified as stage III periodontitis while no patients with clinical periodontal health was observed in the present study. According to the given distribution, to compare the collected data we reassembled participants into two groups as localized periodontitis and generalized periodontitis as shown in Table 1. The result demonstrated that all general demographic and metabolic variables were not significantly different, exception for slightly higher frequency of alcohol consumption in generalized periodontitis group. MetS patients with generalized periodontitis demonstrated significantly greater proportion of severe CAD events than those with localized periodontitis (39% vs. 10%, *p* <  0.05) (Table 1).

**Table 1:** Demographic data, metabolic syndrome and periodontal parameters of two groups according to periodontal status

Variable	Localized periodontitis (***n =*** 48)	Generalized periodontitis (***n =*** 98)	***p***-value
Age (years)	62.25 ± 7.8	63.60 ± 7.9	0.33
Gender			0.1
Male	18 (37.5)	51 (52.0)	
Female	30 (62.5)	47 (48.0)	
Alcohol consumption			**0.049**
Never	**38 (79.2)**	**65 (66.3)**	
Occasionally	**7 (14.6)**	**31 (31.6)**	
Frequently	**3 (6.3)**	**2 (2.0)**	
Smoking			0.22
Never	34 (70.8)	55 (56.1)	
Former	12 (25.0)	35 (35.7)	
Current	2 (4.2)	8 (8.2)	
Diabetes mellitus type 2			0.1
No	11 (22.9)	12 (12.2)	
Yes	37 (77.1)	86 (87.8)	
Metabolic parameters			
BMI (kg/m^2^)	28.31 ± 6.34	27.99 ± 6.93	0.66^a^
Waist circumference (cm)	96.01 ± 13.88	96.24 ± 12.59	0.75^a^
FBS (mg/dL)	110.79 ± 57.79	102.19 ± 49.68	0.92^a^
HbA1C	6.88 ± 1.10	7.0 ± 1.37	0.81 ^a^
LDL-C (mg/dL)	86.21 ± 46.08	83.59 ± 44.58	0.73 ^a^
HDL-C (mg/dL)	46.53 ± 22.39	40.99 ± 22.69	0.10 ^a^
Triglyceride (mg/dL)	112.09 ± 53.79	129.49 ± 63.99	0.09 ^a^
Systolic blood pressure (mmHg)	136.28 ± 16.52	138.16 ± 18.41	0.56
Diastolic blood pressure (mmHg)	75.98 ± 10.04	74.67 ± 9.81	0.46
MetS components: n (%)			
FBS ≥ 110 mg/dL	22 (45.83)	57 (58.16)	0.20
SBP ≥ 130 or DBP ≥ 85 mmHg	43 (89.6)	87 (88.8)	0.88
Triglycerides ≥150 mg/dL	10 (20.83)	24 (24.5)	0.67
Low HDL	31 (64.6)	72 (73.5)	0.27
High WC	41 (85.4)	78 (79.6)	0.39
Periodontal status (± SD)			
Number of teeth	**24.48 ± 3.6**	**19.51 ± 6.94**	**< 0.001**^a^
Mean PPD (mm.)	**2.44 ± 0.27**	**2.91 ± 0.61**	**< 0.001**^a^
Mean CAL (mm.)	**2.91 ± 0.36**	**4.34 ± 1.28**	**< 0.001**^a^
GI	**1.26 ± 0.28**	**1.50 ± 0.43**	**0.001**^a^
PI	**1.28 ± 0.30**	**1.68 ± 0.54**	**< 0.001**^a^
%Tooth with PPD ≥ 5 mm	**4.40 ± 6.90**	**25.08 ± 26.07**	**< 0.001**^a^
%Tooth with interdental CAL ≥ 5 mm	**14.10 ± 13.67**	**61.71 ± 26.70**	**< 0.001**^a^
History of severe CAD: n (%)			
No	**38 (79.2)**	**59 (60.2)**	**0.02**
Yes	**10 (20.8)**	**39 (39.8)**	
FGF21 level (ng/dl)	**231.79 ± 160.47**	**432.03 ± 398.25**	**0.001**^a^

Data were presented as mean ± SD or n (%). Bold values present significant difference (*P <* 0.05). Categorical data using Chi-square.

^a^ = mean comparison using Mann-witney U-test (Unpaired T-test for the other variables)

For the validation of serum FGF21 level, patients with generalized periodontitis presented the significantly higher level of FGF21 level when compared to localized periodontitis group (Table 1). The correlation between FGF21 and periodontal parameters including number of teeth, mean CAL and %tooth with CAL ≥ 5 mm were significant (Table 2). Furthermore, both groups showed the correlation between FGF21 level and hyperglycemia, hypertriglyceridemia and lowered HDL which were metabolic components that were significantly correlated to FGF21 level (Table 3).

**Table 2:** Correlation between FGF21 level and periodontal parameters

	Group		% Pl	% GI	Number of teeth	Mean PPD	Mean CAL	% tooth with PPD ≥ 5	% tooth with interdental CAL ≥ 5
**FGF21 level (ng/dl)**	**All**	r	0.06	0.13	**−0.21 **	0.05	**0.24 **	0.08	**0.27**
		P	0.50	0.12	**0.01**	0.54	**0.003**	0.35	**0.001**
	**Localized periodontitis**	r	0.06	−0.11	0.02	−0.004	0.039	−0.113	0.02
		P	0.70	0.47	0.88	0.98	0.79	0.45	0.91
	**Generalized periodontitis**	r	−0.04	0.15	−0.17	−0.08	0.14	−0.01	0.15
		P	0.72	0.15	0.10	0.43	0.17	0.91	0.14

All correlation analyzed by Spearman rank test. Bold values present significant difference (*P <* 0.05)

**Table 3:** Correlation between FGF21 level and metabolic components

	Group		FBS	BP	TG	HDL	WC
**FGF21 level (ng/dl)**	**All**	r	**0.18**	0.11	**0.29**	**0.18**	0.05
		P	**0.03**	0.20	**< 0.001**	**0.03**	0.56
	**Localized periodontitis**	r	0.16	0.12	**0.38**	−0.01	0.18
		P	0.28	0.43	**0.01**	0.94	0.21
	**Generalized periodontitis**	r	0.17	0.13	**0.27**	**0.24**	0.016
		P	0.11	0.21	**0.01**	**0.02**	0.88

All correlation analyzed by Spearman rank test. Bold values present significant difference (*P <* 0.05). **Abbreviation:***FBS* Fasting blood sugar ≥100 mg/dl or treated, *BP* Systolic or diastolic blood pressure ≥ 130/85 mmHg or treated, *TG* Triglyceride ≥150 mg/ dl or treated, *HDL* High density lipoprotein cholesterol < 40 mg/dl in men and < 50 mg/dl in women, *WC* Waist circumference ≥ 90 cm in men and ≥ 80 cm in women

Finally, the multivariate model demonstrated the positive association between FGF21 and generalized periodontitis with an odds ratio of 27.12 (95% CI: 5.24–140.35), independently on age, smoking, alcohol consumption, and metabolic disturbance, as shown in Table 4.

**Table 4:** Multiple logistic regression showing the association between FGF21 level and generalized periodontitis

				FGF21 level^**a**^			
**Analysis outcome**	***B***	**SE**	**Wald**	**Crude odds ratio (95% C.I.)**	***P*-value**	**Adjusted odds ratio**^**¶**^**(95% C.I.)**	***P*-value**
**Localized periodontitis (control)**				1.00		1.00	
**Generalized periodontitis**	3.30	0.84	15.48	**9.71 (2.71–34.91)**	**< 0.001**	**27.12 (5.24–140.35)**	**0.012**

^¶^ Model mutually adjusted for age, gender, smoking, alcohol consumption, diabetic status, presence of component FBS, TG, HDL. ^a^ = Log transformed; *B* = regression coefficient; SE = standard error; Wald = Wald test

## Discussion

This is the first study reporting the alterations of serum FGF21 levels in MetS patients with periodontitis. The major findings showed that MetS patients with generalized periodontitis demonstrated a significantly higher level of serum FGF21 than those with localized periodontitis. Moreover, the correlation between FGF21 level and periodontal parameters including Number of remaining teeth and CAL was observed. The level of serum FGF21 was also associated with the presence of generalized periodontitis after adjusting of confounding factors.

FGF21 is a hormone primarily produced by the liver which plays a potential role in regulation of metabolic processes, especially in improving glucose level, insulin activity and lipid metabolism [3]. Previously, the animal and human studies have consistently proposed this protein as a therapeutic biomarker for MetS [10–12, 20, 24, 25]. The finding of this study presented that metabolic parameters correlated with FGF21 level were hypertriglyceridemia and low HDL-C, which was in line with the previous reports [26–28]. In mice model, the study showed that FGF21 is able to lower plasma triglycerides via regulating CD36 which results in stimulating TG uptake into adipose tissue [29]. Likewise, obesity and diabetic patients who received human recombinant FGF21 for 4 weeks demonstrated improved level of HDL-C [24]. Therefore, it was possibly implied that individuals who had periodontitis might comprise a higher chance of dyslipidemia development, which subsequently increased an expression of FGF21 level as a protective role.

In addition to metabolic parameters, the main outcome of the current study demonstrated a higher level of serum FGF21 in generalized periodontitis group, when compared to group with localized periodontitis. It was in concordance with the previous report conducted in Cairo [18]. The findings suggested that the elevation of FGF21 in GCF might act as a protective mechanism to oppose periodontal inflammation in diabetic patients with periodontitis. This was confirmed by the reduction of FGF21 level following periodontal treatment. The anti-inflammation effect of FGF21 was also evident by Li and colleagues in mice model [30], in which the expression of FGF21 was responsible for LPS-induced inflammation through inhibiting IL-1β expression in NF-κB pathway and enhancing elevation of IL-10.

Furthermore, our findings presented that periodontal parameters including CAL and number of remaining teeth were significantly correlated with FGF21 level. It was obvious that CAL represents the past destruction of periodontal tissues which is one of the main clinical features for periodontitis related to tooth loss. Although there have been no studies focusing on FGF21 and periodontal parameters before, the association between these parameters and MetS have been widely established [31–34]. The evidence showed that CAL was significantly associated with increased C-reactive protein, a major biomarker representing the state of systemic inflammation which mainly contributed to metabolic disturbance [35]. It was thus possible that the elevation of serum FGF21 level might be a result of its anti-inflammation effect responsive to systemic inflammation induced by generalized periodontitis. Additionally, the latest meta-analysis highlighted that MetS patients were more likely to has lesser number of remaining teeth, suggestive the negative effect on functional dentition and fiber intake. A worsen chewing function might lead patients to unavoidably take more high cholesterol foods and finally develop metabolic disorder [34]. In this context, increased serum FGF21 might be an indirect consequence of a compensatory up-regulation against a presence of dyslipidemia components as shown as the correlation analysis in this study.

Conversely, the other two studies validating serum FGF21 in diabetic patients with periodontitis presented the contradictory results [19, 20]. They found that serum FGF21 level was increased after periodontal treatment and negatively correlated with improved HbA1c. These was presumed that alteration of serum FGF21 was mainly attributed to glycemic controls which was beneficially affected by reduction of periodontal inflammation. Nevertheless, these studies had small sample size and no periodontal control group to compare.

Finally, the multiple logistic regression analysis confirmed the association between FGF21 and presence of generalized periodontitis (OR = 27.12) even after adjusting confounding variables including age, gender, smoking, alcohol consumption, diabetic status, presence of components FBS, TG, HDL. Therefore, it was possible that the altered expression of serum FGF21 might be also modified by the systemic inflammation induced by generalized periodontitis apart from metabolic abnormalities. However, a true linking mechanism explaining this phenomenon is still not fully understood.

There were some limitations in the current study. First, the outcomes from this cross-sectional study could not be explained in causal-effect direction between FGF21 and periodontitis in MetS patients. Longitudinal study or clinical controlled trials investigating FGF21 before and after periodontal treatment might clarify the role of periodontitis to FGF21. Secondly, MetS components or medications might affect the level of FGF21. The future study with healthy control group would clarify these confounders. Thirdly, the older population can affect both periodontal status and serum FGF21 level so a different age group is still needed to be investigated. Lastly, the biomarkers such as TNF-α and IL-1β, which are targeted by FGF21 and involved in periodontitis development, were not determined in the present study. Therefore, the further studies about relationship between FGF21 and periodontitis is still needed to be clarified.

## Conclusions

The elevation of serum FGF21 might be a potential biomarker for MetS patients who have risk of generalized periodontitis. Several parameters including CAL, number of teeth, and dyslipidemia demonstrated a correlation to serum FGF21 level. Although the present study was not able to fully answer the relationship between FGF21 and periodontitis, the data elucidates the significant effect of periodontal disease on systemic health, especially MetS. Periodontal examination and therapy should be included as a part of treatment for MetS patients.

## Data Availability

The datasets generated and/or analysed during the current study are not publicly available due to their containing information that could compromise the privacy of research participants but are available from the corresponding author on reasonable request.

## Abbreviations

FGF21: Fibroblast growth factor 21
MetS: Metabolic syndrome
HDL-C: High-density lipoprotein cholesterol
FBS: Fasting blood sugar
CVD: Cardiovascular disease
GCF: Gingiva crevicular fluid
ELISA: Enzyme-linked immunosorbent assay
LDL-C: Low-density lipoprotein cholesterol
PI: Plaque index
GI: Gingival index
PPD: Probing pocket depth
CAL: Clinical attachment loss
CAD: Coronary artery disease
IL: Interleukin
